# High-dose fluconazole in combination with amphotericin B is more efficient than monotherapy in murine model of cryptococcosis

**DOI:** 10.1038/s41598-017-04588-7

**Published:** 2017-07-05

**Authors:** Julliana Ribeiro Alves Santos, Noelly Queiroz Ribeiro, Rafael Wesley Bastos, Rodrigo Assunção Holanda, Letícia Chagas Silva, Estela Rezende Queiroz, Daniel Assis Santos

**Affiliations:** 1 Mestrado em Biologia Parasitária – Universidade CEUMA (UNICEUMA), São Luís, Maranhão Brazil; 2 Mestrado em Meio Ambiente – Universidade CEUMA (UNICEUMA), São Luís, Maranhão Brazil; 3 Departamento de Microbiologia, Instituto de Ciências Biológicas, Universidade Federal de Minas Gerais, Belo Horizonte, Minas Gerais Brazil; 4 Departamento de Química, Universidade Federal de Lavras (UFLA), Campus 17 Universitário, Lavras- MG, Brazil

## Abstract

*Cryptococcus* spp., the causative agents of cryptococcosis, are responsible for deaths of hundreds of thousands of people every year worldwide. The drawbacks of available therapeutic options are aggravated by the increased resistance of yeast to the drugs, resulting in inefficient therapy. Also, the antifungal 5FC is not available in many countries. Therefore, a combination of antifungal drugs may be an interesting option, but *in vitro* and theoretical data point to the possible antagonism between the main antifungals used to treat cryptococcosis, i.e., fluconazole (FLC), and amphotericin B (AMB). Therefore, *in vivo* studies are necessary to test the above hypothesis. In this study, the efficacy of FLC and AMB at controlling *C. gattii* infection was evaluated in a murine model of cryptococcosis caused by *C. gattii*. The infected mice were treated with FLC + AMB combinations and showed a significant improvement in survival as well as reduced morbidity, reduced lung fungal burden, and the absence of yeast in the brain when FLC was used at higher doses, according to the Tukey test and principal component analysis. Altogether, these results indicate that combinatorial optimization of antifungal therapy can be an option for effective control of cryptococcosis.

## Introduction

Fungal diseases are responsible for the deaths of more than one million people each year around the world^1, 2^, and a half of these deaths are caused by cryptococcosis: this mortality is higher than the mortality caused by tuberculosis and similar to that caused by malaria^3^.

*Cryptococcus neoformans* and *C. gattii* are the main etiological agents of cryptococcosis; they affect immunosuppressed and immunocompetent individuals, respectively^4^. The infection starts with inhalation of fungal propagules from the environment^5^. Upon reaching the respiratory tract, the yeast cause pneumonia and can disseminate to the central nervous system (CNS), causing the most severe form of the disease: meningoencephalitis^4–6^.

Cryptococcosis is fatal without effective treatment, and anticryptococcal therapy is currently a concern because there are few suitable (and available) drugs. At the same time, therapeutic failure can be caused by an increase in resistance to antifungal drugs^7^. The current standard of care is based on three antifungals: fluconazole (FLC), amphotericin B (AMB), and 5-flucytosine (5-FC)^8^. The use of AMB in combination with 5-FC is known as the “gold standard” because they can act in synergy against the pathogen. In contrast, AMB and 5-FC are not available in all countries and are nephrotoxic and hepatotoxic^9^, respectively; this situation restricts the therapeutic options. Besides, AMB administration requires hospitalization. Regarding FLC, despite its low toxicity and availability in many countries, the resistance of *Cryptococcus* to this drug is increasing^10, 11^. Treatment of cryptococcosis with FLC and other azole drugs usually is lengthy; this state of affairs is also a concern because *C. neoformans* and *C. gattii* are believed to be intrinsically heteroresistant to these drugs^12–14^.

In this context, the development of new therapies for cryptococcosis is necessary. Nevertheless, the long time spent on the research and development of a new drug, the high cost, and the lack of incentives and interest on the part of the government authorities and industries, make the development of new drugs - and their introduction into clinical practice - very difficult^15, 16^. Although the number of deaths caused by cryptococcosis is high, the proportion of biomedical funding allocated to this area in 2014 was only 0.3% low when compared to other diseases characterized as neglected (amount of four funding - US NIH,UK MRC, Australian NHMRC and the Wellcome Trust)^2^. Therefore, one viable option is a search for alternative treatments based on the antifungals already available. Given the greater difficulty with the use of 5-FC, the simultaneous combination of AMB and FLC is interesting. The fungicidal effect of polyene associated with the easy administration of the azole, makes this combination a promising modality. Although previous study has shown synergism or additive effect between amphotericin B associated to voriconazole, and indifference or neutral effect or even antagonism for this combination^17, 18^, *in vivo* tests of these drugs in combination are scarce. The aim of this study was to evaluate the *in vivo* efficacy of the combination AMB + FLC using different doses of FLC in a murine model of cryptococcosis caused by *C. gattii*. Briefly, 150 mg/kg FLC combined with 0.5 mg/kg AMB increased the survival of mice, reduced the morbidity, decreased the fungal load in the lungs, and inhibited yeast growth in the brain.

## Results

### High-dose FLC plus AMB prolonged survival of the animals infected with *C. gattii*

**Figure 1:**
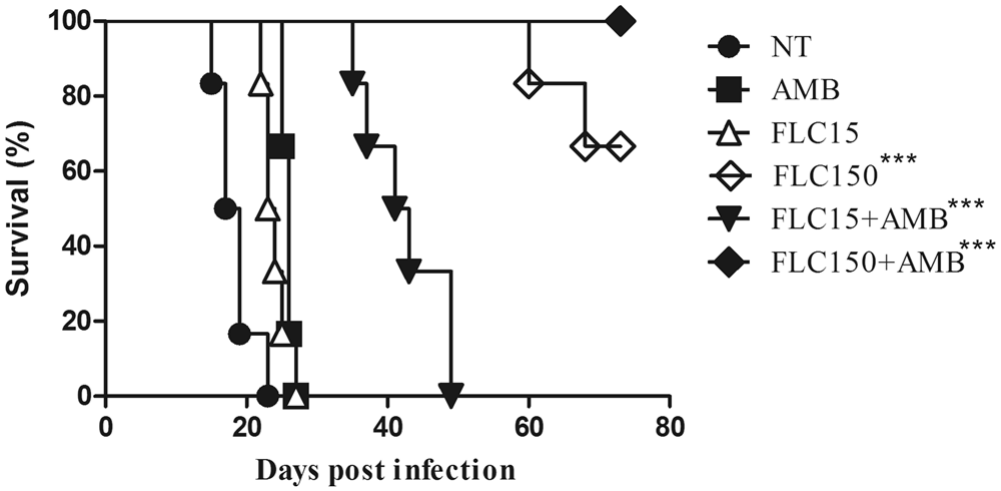
Six mice per group were inoculated by the intratracheal route with 1 × 10^6^ cells of L27/01 cryptococcal cells. Fluconazole (15 or 150 mg/kg/day) and amphotericin B (0.5 mg/kg/day) alone or in combination were administered intraperitoneally (i.p.) from one day post-infection. Animals were monitored daily for the survival curve. All treatments significantly prolonged the median survival of mice infected. Treatment with 150 mg/kg/day fluconazole alone or in combination with amphotericin B and 15 mg/kg/day fluconazole in combination with amphotericin B improved the median survival of mice treated with antifungals in monotherapy. NI (not infected); NT (Control, untreated), AMB (Amphotericin B 0.5 mg/kg/day), FLC15 (Fluconazole 15 mg/kg/day), FLC150 (Fluconazole 150 mg/kg/day) FLC15 + AMB (Fluconazole 15 mg/kg/day + Amphotericin B 0.5 mg/kg/day), FLC150 + AMB (Fluconazole 150 mg/kg/day + Amphotericin B 0.5 mg/kg/day). ***P < 0.001 (compared with the NT group).

A Kaplan-Meier curve was used to determine the survival rates (Fig. 1). The median survival in the nontreated (NT) group was 18 days, whereas the median survival was 23.5, 26, and 42 days for the groups treated (P < 0.05) with low-dose FLC (15 mg/kg/day), AMB alone, or AMB plus low-dose FLC, respectively. Although 33.33% of the animals died within 80 dpi, increased survival was observed in animals treated with high-dose FLC (150 mg/kg/day) as compared to the NT group or the other groups (P < 0.05). Of note, all animals survived during the treatment with high-dose FLC in combination with AMB.

### High-dose FLC in combination with AMB efficiently reduced the fungal burden

**Figure 2:**
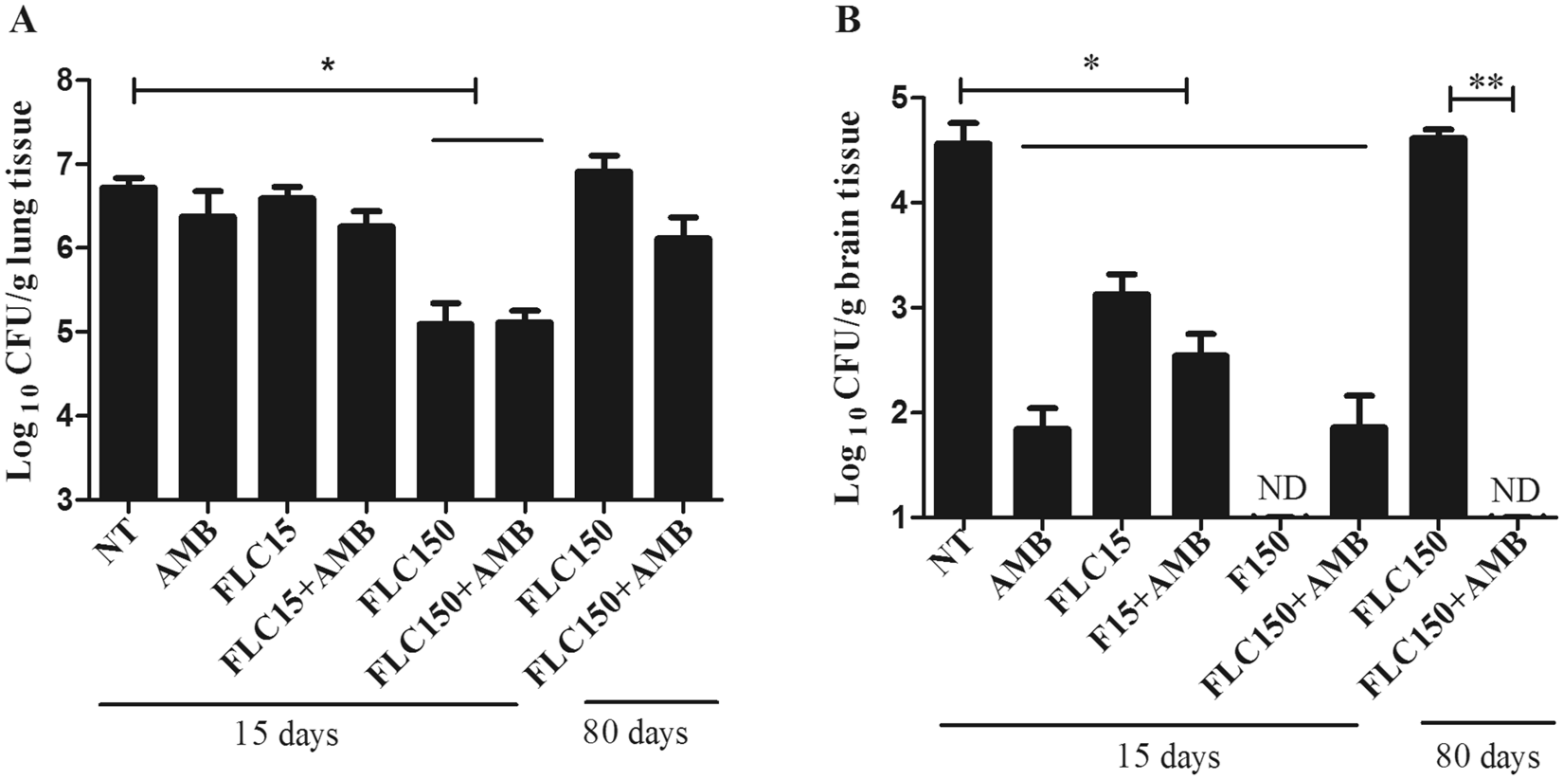
Mice were euthanized 15 or 80 days post-treatment. Lungs (**A**) and Brain (**B**) were removed and processed for measurement of fungal burden. Animals infected and treated with fluconazole 150 alone or in combination showed significant reduction of Log_10_ CFU/g in the lungs, but no significant reduction of fungal burden in lungs occurred in other groups (**A**). NT (Control, untreated), AMB (Amphotericin B 0.5 mg/kg/day), FLC15 (Fluconazole 15 mg/kg/day), FLC150 (Fluconazole 150 mg/kg/day) FLC15 + AMB (Fluconazole 15 mg/kg/day + Amphotericin B 0.5 mg/kg/day), FLC150 + AMB (Fluconazole 150 mg/kg/day + Amphotericin B 0.5 mg/kg/day), ND: not detected. *P < 0.05 **P < 0.01.

The fungal burden in the lungs (Fig. 2A) at 15 dpi was not reduced significantly (P > 0.05) by low-dose FLC and AMB, alone or in combination. Treatment with high-dose FLC alone or in combination with AMB was significantly (P < 0.05) effective in reducing the fungal burden in the lungs of the animals compared to the NT group (Fig. 2A). On the other hand, at 80 dpi, the fungal burden in mice treated with high-dose FLC was significantly reduced only when FLC was tested in combination (Fig. 2A).

Regarding the brain, the fungal burden (Fig. 2B) was significantly (P < 0.05) reduced on day 15 postinfection by treatment with FLC alone or in combination, regardless of the concentrations tested. Furthermore, at 15 dpi, there was no recovery of viable colonies from the brain of mice in the group treated with high-dose FLC alone (P < 0.05) and on day 80 post infection in group high-dose FLC + AMB (P < 0.05; Fig. 2B).

### High-dose FLC combined with AMB improved the behavior of mice

**Figure 3:**
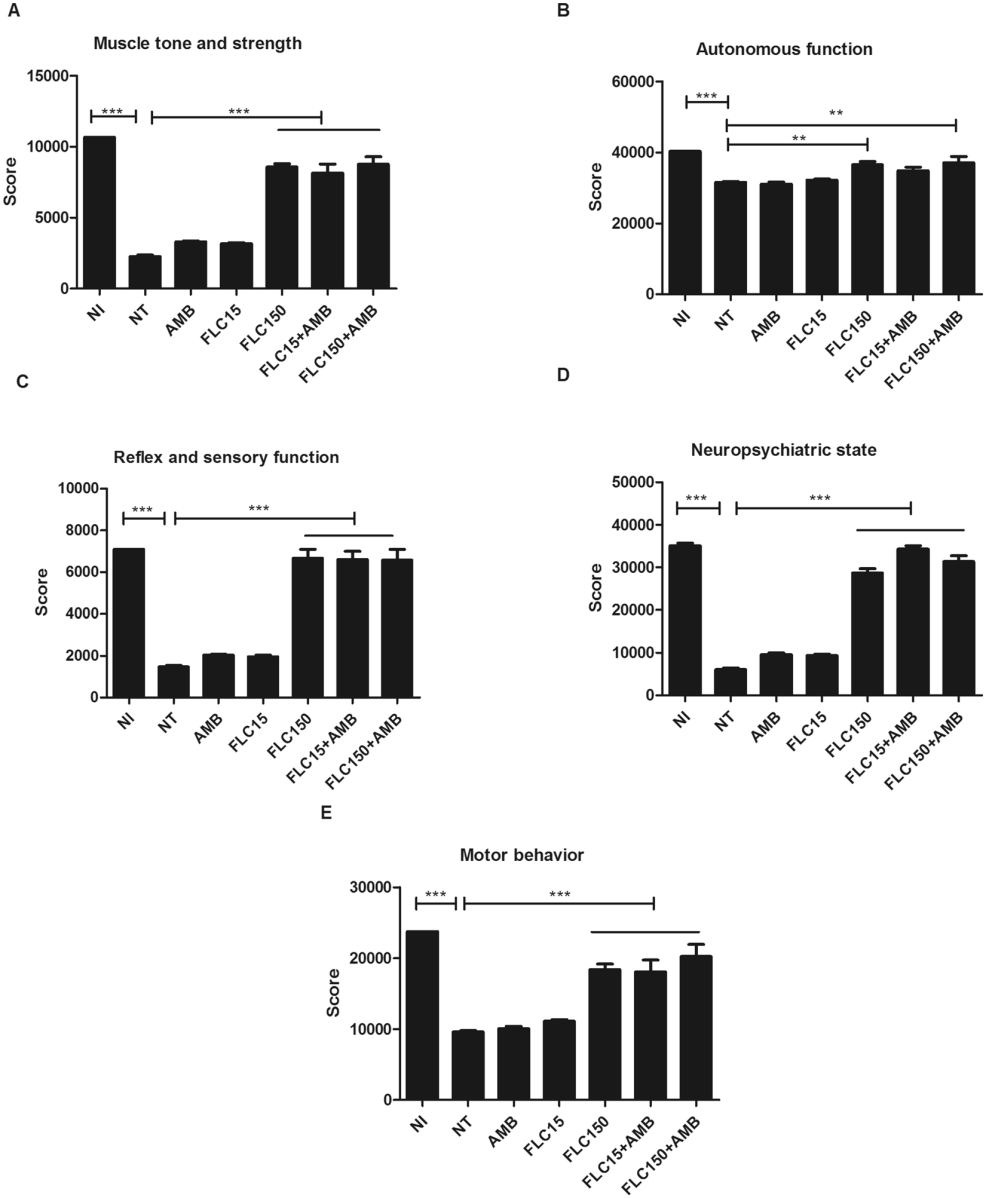
Behavioral profile evaluation (SHIRPA protocol) of animals infected with *C. gattii* and treated with amphotericin B (AMB), fluconazole (FLC15 and FLC150) and the combination FLC15 + AMB and FLC150 + AMB (**A–E**). (**A**) Muscle tone and strength; (**B**) autonomous function; (**C**) Reflex and sensory function (**D**) neuropsychiatric state; (**E**) motor behavior. NI (not infected); NT (Control, untreated), AMB (Amphotericin B 0.5 mg/kg/day), FLC15 (Fluconazole 15 mg/kg/day), FLC150 (Fluconazole 150 mg/kg/day) FLC15 + AMB (Fluconazole 15 mg/kg/day + Amphotericin B 0.5 mg/kg/day), FLC150 + AMB (Fluconazole 150 mg/kg/day + Amphotericin B 0.5 mg/kg/day). **P < 0.01 ***P < 0.001 (difference when compared to NT).

The results revealed significant behavioral changes (P < 0.05) in groups FLC15 and AMB (Fig. 3). In groups FLC150, FLC15 + AMB, and FLC150 + AMB the respective treatment improved the behavior of the animals, in terms of muscle tone and strength – MF (Fig. 3A), reflex and sensory functions - RF (Fig. 3C), neuropsychiatric state - NS (Fig. 3D), and motor behavior – MB (Fig. 3E). In contrast, the autonomous function - AF (Fig. 3B) was not improved in mice of group FLC15 + AMB (P > 0.05).

**Figure 4:**
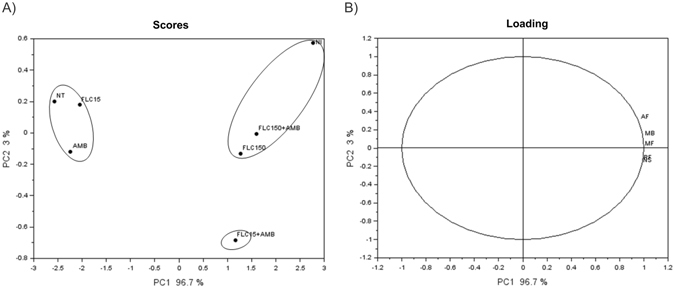
Graphic representation of the scores (**A**) and loadings (**B**) of the analyzes performed on the animals of the groups. NI (not infected); NT (Control, untreated), AMB (Amphotericin B 0.5 mg/kg/day), FLC15 (Fluconazole 15 mg/kg/day), FLC150 (Fluconazole 150 mg/kg/day) FLC15 + AMB (Fluconazole 15 mg/kg/day + Amphotericin B 0.5 mg/kg/day), FLC150 + AMB (Fluconazole 150 mg/kg/day + Amphotericin B 0.5 mg/kg/day) at the end of 42 days of treatment, evaluated in relation to the axes defined by the main components (PC1 and PC2). NS: neuropsychiatric state, MB: motor behavior, AF: autonomic function, MF: muscle tone and strength, and RF: reflex and sensory function.

PCA, when applied to the data, explained 99.7% of total variance in the characteristics of the animals subjected to the different treatments. The first component (PC1) is responsible for 96.7% of the data matrix variance and horizontally separates treatments NI (not infected) and NT (Fig. 4A). Thus, we can say that there is differentiation of treatment in three groups—NI, FLC150 + AMB, and FLC150—because they showed higher AF, MB, and MF, thereby each of the three distanced itself from the other groups, especially from NT, AMB, and FLC15. The second component (PC2) discriminates, vertically, group NI from group FLC15 + AMB, by autonomic function and neuropsychiatric status, explaining 3% of the total variance. The loadings characterize the trends among the analyzed variables (Fig. 4B). Along the PC2 axis, the variables that most influenced this component are, vertically, AF and MB (with positive values): the main variables for animals of groups NI, FLC150 + AMB, and FLC150. The other variables that most influenced this component (with negative values) are NS and RF: the main variables for group FLC15 + AMB.

The results from the correlation matrix for the analyzed variables are presented in the form of correlation table, containing the calculated pair correlation coefficient and the indicator of the strength of correlation between all pairs of variables^19^. Thus, the correlation coefficients between MF, AF, RF, NS e MB were evaluated to study the interrelationship between them (Table 1). A strong positive correlation was recorded between all behavioral measurements.

**Table 1:** Correlation coefficients among behavioral profile (SHIRPA protocol) of animals infected with C. gattii and treated with amphotericin B (AMB), fluconazole (FLC15 and FLC150) and the combination FLC15 + AMB and FLC150 + AMB. MF: muscle tone and strength, AF: autonomic function, RF: reflex and sensory function, NS: neuropsychiatric state and MB: motor behavior.

	MF	AF	RF	NS	MB
MF	1.0000	0.95522	0.9846	0.9777	0.9901
AF		1.0000	0.9004	0.8869	0.9807
RS			1.0000	0.9906	0.9595
NS				1.0000	0.9579
MB					1.0000

In summary, the above analyses showed that groups FLC150 and FLC150 + AMB were the closest to normality, presenting clinical signs similar to those in the NI group. On the other hand, groups AMB and FLC15 showed profiles similar to the profile of the NT group. The FLC15 + AMB group showed an intermediate clinical profile.

## Discussion

FLC and AMB remain as the main options for the treatment of cryptococcosis in many countries, as in Brazil, especially due the unavailability of 5FC, compromising the establishment of the “gold standard” therapy^20–23^. This fact lead us to conduct this study and makes possible the suggestion of the combination of FLC and AMB as induction therapy in countries where 5FC is not available. The scarce alternatives for cryptococcosis treatment are stimulating research into new therapies in the form of antifungal combinations. Unfortunately, there is a controversy regarding the interaction of FLC and AMB as treatments of fungal infections^24–26^. Day *et al*.^26^ studied clinical efficacy of the combination of AMB and FLC. This treatment did not confer a survival advantage, as compared with monotherapy. However, the parameters analyzed were different from our study: the patients were immunosuppressed and infected with *C. neoformans*, in addition, the dose used of the drugs were not same^26^. Furthermore, combination tests performed by our group revealed that the interaction of FLC and AMB against *C. gattii* strains, in general, is indifferent (as opposed to additive) *in vitro* ^27^. Nevertheless, at some concentrations of the drugs, we observed a synergistic and antagonistic interaction of FLC and AMB against *C. gattii* ^27^. These results are in agreement with recent studies indicating that the combination of FLC and AMB may be indifferent but also antagonistic or synergistic against other species of *Cryptococcus* ^18^. Interestingly, Mukherjee *et al*., 2005 proposed two theories of interaction between fluconazole and amphotericin B: 1. Depletion, where azole associated to polyene resulting in antagonism, since azole depletes the ergosterol of fungal cells, reducing the targets for the polyene. 2. Enhancement, where synergism is observed, since polyenes, by pore formation, facilitate the entry of azoles to the intracellular space and the action in inhibiting the ergosterol biosynthesis^25^.

In the present study, we tested low-dose FLC (15 mg/[kg.day], corresponding to 80 mg/day in humans) and high-dose FLC (150 mg/[kg.day], corresponding to 800 mg/day in humans) alone or in combination with AMB (0.5 mg/kg/day) to elucidate the controversy regarding this combination and cryptococcal infections. FLC at (150 mg/kg/day) and the combination of AMB and FLC dose-independently prolonged the survival of our mice. In contrast, FLC (150 mg/kg/day) as monotherapy or in combination was found to be effective at reducing the fungal burden in the lungs of the infected animals compared to nontreated animals. Although high-dose FLC monotherapy prolonged the survival of the animals, the first mice died at ~60 dpi. This is probably because at 80 dpi, the fungal burden was elevated in the brain^28^. It is known that the brain yeast clearance is related to the therapeutic outcome. However it is important to consider a limitation of the plating method for determining the fungal burden, since the lower limit of detection of this technique is 10 CFU/g^29^. Nonetheless, the combination with high-dose FLC was better at reducing the fungal burden in the lungs and brain, alleviating morbidity of the animals without deaths during the 80 days evaluated. The combination of high-dose FLC and AMB was effective at brain clearance of yeast or at preventing the fungal translocation from lungs to the brain.

Compared to the univariate techniques, the multivariate analysis such as PCA represents a powerful tool for exploring large datasets extracted from biological systems which contain multiple variables and which may contain missing data points^19, 30, 31^, and its application for evaluation of treatment efficacy is highly recommendable^19, 32, 33^.

Judging by the PCA and behavioral data, our results indicate that formulations FLC150 + AMB and FLC150 are the most efficient in the control of *C. gattii* infections in mice. PCA has the special characteristic of capturing the key components of an assay, placing them in linear space and linking redundant information to them. In qualitative analysis, PCA plays two important roles^34^. First, PC scores can graphically present the structure of original data in two- or three-dimensional space, which may show groups of observations or trends. Second, PCA is often coupled with pattern recognition methods for classification purposes^35^.

Thus, chemometric methods such as PCA can provide a comprehensive view and thus help to examine the effects of antifungal treatments on cryptococcosis throughout the experimental process as well as to discover possible correlations and the *in vivo* efficacy of therapies, such as the combination AMB + FLC in our murine model of cryptococcosis caused by *C. gattii*. Our results show that the animals of groups FLC150 and FLC150 + AMB were clinically better off, being close to the NI group. It is worth noting the arrangement of the variables along PC1, where the loadings revealed consistent relations between the analyzed variables and treatments FLC150 + AMB and FLC150. The loadings, which exhibit the parameters evaluated (MF, RF, NS, MB and AF), also show separation of these treatments from NT, AMB, and FLC15. In addition, our data suggest that formulation FLC15 + AMB has a potential for further studies: this conclusion can be deduced from the proximity of this treatment, horizontally and throughout PC1, with the FLC150 treatment. The results of correlation analysis are consistent with the results of PCA and are also in agreement with the expected relationships between some descriptors, previously cited^19, 32, 33, 36–38^.

In conclusion, considering the promising results on the combination high-dose FLC + AMB, we propose that further studies including clinical research are needed to identify more accurately an optimal treatment of cryptococcosis with the two most widely used antifungals worldwide.

## Methods

### Animal protocol

The protocol for animal experiments was approved by the Comissão de Ética no Uso de Animais (CEUA) from the Universidade Federal de Minas Gerais, Brazil (protocol 366/2013) and animal experiments were performed in strict accordance with the Brazilian Federal Law 11,794 establishing procedures for the scientific use of animals. All mice were housed in clean bedding (five mice per cage) with food and water *ad libitum* in a controlled environment with a 12 h light/dark cycle at 23 °C. C57BL/6 male mice (6 to 8 weeks old) were used in all the experiments. The mice were anesthetized with intraperitoneal (i.p.) injection of ketamine (80 mg/kg) and xylazine (10 mg/kg) in sterile saline, then inoculated with intratracheal (i.t.) injection of 30 μL of the L27/01(UFMG-M-Y6141) *C. gattii* strain at 10^6^ colony-forming units (CFUs) per animal or PBS (not infected [NI] group).

### Antifungal therapy

After the infection by inoculation, the mice were subdivided into six groups, six animals each, as follows: (i) nontreated (NT), (ii) received AMB (0.5 mg/kg·day) (Sigma-Aldrich, St. Louis, MO), (iii) received low-dose FLC (15 mg/kg·day) (Sigma-Aldrich), (iv) received high-dose FLC (150 mg/[kg·day]), (v) received low-dose FLC plus AMB (0.5 mg/[kg·day]), and (vi) received high-dose FLC plus AMB (0.5 mg/[kg·day]). Another control group was used: animals not infected but treated with PBS (NI). Treatments were initiated at 24 h after infection and were administered by i.p. injection, once daily, until mice appeared moribund in survival experiments. The mice were monitored twice daily for survival curve data. All the experiments were performed three times to confirm the data and the results were always reproducible.

### Behavioral analysis

The behavioral and functional assessment of neurological diseases was conducted by the SmithKline/Harwell/Imperial College/Royal Hospital/Phenotype Assessment (SHIRPA) protocol, as previously described^39, 40^. The assay evaluated five functional categories: neuropsychiatric state, motor behavior, autonomic function, muscle tone, and strength as well as reflex and sensory function. The score in each functional category was obtained using the EpiData 3.1 software^39, 40^.

### Determination of fungal burden (CFUs)

After analysis of the survival curve, other groups of mice were infected by i.t. injection and the treatment continued until day 15 or day 80. The animals were euthanized at 15 or 80 days postinfection (dpi) to collect the lungs and brain. The organ homogenates were plated onto saboraud dextrose agar SDA for 48 h at 35 °C to determine the fungal burden expressed in CFUs per gram of tissue.

### Statistical analyses

Software GraphPad Prism, version 5.0, for Windows (GraphPad Software, San Diego, CA, USA) was used for all the statistical analyses, with P < 0.05 assumed to denote statistical significance. The survival curve was plotted by the Kaplan-Meier method, and the results were analyzed by the logrank test. The results on CFUs were analyzed by analysis of variance (ANOVA) and the nonparametric Friedman test. SHIRPA data were analyzed using area under the curve, ANOVA, and Tukey’s test. The experiment was conducted in a completely randomized design (CRD) in a factorial scheme (7 × 6), i.e., seven treatments with six mice each.

Additionally, the SHIRPA data were processed in the SCILAB 5.5.2 software, and principal component analysis (PCA) was performed from the correlation matrix. PCA is a chemometric multivariate analysis method used to transform multidimensional data into low-dimension data while maintaining the linear relations of pairwise distances^41^. By means of the PCA model, the data are decomposed into a set of a few orthogonal latent variables, called principal components (PCs), defining a new coordinate system and the so-called loadings, describing the contribution of individual variables to a given PC^34^. The PCA method, from the correlation matrix, consists of transforming a set of variables Z1, Z2, …, Zp into a new set of variables Y1 (PC1), Y2 (PC2), …, Yp (PCp). Thus, a new set of p variables not correlated with each other and arranged in a decreasing order of variances is defined^19, 42^. According to Budaev^43^, PCA is often used in animal behavior research, for this: (1) correlations between the original behavioural measures are calculated; (2) the correlation matrix is subjected to specific transformations, resulting in a new set of linear combinations of the original measures (principal components); (3) loadings of the original measures on these principal components are calculated, which represent correlations between the original measure and the principal components^43^. Usually, the first two PCs contain most of the variance of the data and can substitute the original more numerous variables, since one can sensibly discard the other components, which reduces the number of variables. Thus, the interpretation of the results becomes visually simpler, and consequently a better understanding of the all assay is obtained. For this purpose, a PCA model was extracted in order to discern (in the score plot) the treatments that localize near the uninfected group (NI) and to identify (in the loading plot) the behavioral pattern that mainly characterizes them. The correlation matrix for the analyzed variables was inspected in order to explore their relationships. For this, the pair correlation coefficient was calculated.
